# Characteristics, Treatment Complexity, and Outcome of Mixed-Phenotype Acute Leukemia in Children in a Low–Middle-Income Country

**DOI:** 10.3389/fonc.2022.941885

**Published:** 2022-07-07

**Authors:** Maram Salama, Sonia Ahmed, Sonya Soliman, Nahla El-Sharkawy, Sherine Salem, Amr El-Nashar, Reham Khedr, Leslie Lehmann, Iman Sidhom, Alaa El-Haddad

**Affiliations:** ^1^ Pediatric Oncology Department, Children’s Cancer Hospital Egypt (CCHE-57357), Cairo, Egypt; ^2^ Pediatric Oncology Department, National Cancer Institute Cairo University and Children's Cancer Hospital Egypt (CCHE-57357), Cairo, Egypt; ^3^ Clinical Pathology Department, National Cancer Institute Cairo University and Children's Cancer Hospital Egypt (CCHE-57357), Cairo, Egypt; ^4^ Department of Research, Children’s Cancer Hospital Egypt (CCHE-57357), Cairo, Egypt; ^5^ Stem Cell Transplant Center, Dana-Farber/Boston Children’s Cancer and Blood Disorders Center, Boston, MA, United States

**Keywords:** mixed phenotype acute leukemia, pediatrics, low and middle-income countries, *FLT3-ITD*, acute leukemia, malnutrition

## Abstract

**Background:**

Mixed-phenotype acute leukemia (MPAL) in children is an uncommon subtype of acute leukemia that cannot be definitively assigned to a specific lineage. There is no consensus on the best approach to therapy. Management is more complex in low–middle-income countries (LMICs).

**Aim:**

To evaluate the clinicopathological characteristics and outcomes of patients with MPAL in a developing country.

**Patients and Methods:**

A retrospective descriptive study of 42 pediatric patients newly diagnosed with MPAL from July 2007 until December 2017.

**Results:**

The immunophenotyping was T/Myeloid in 24 patients (57.1%) and B/Myeloid in 16 (38.1%). Three subjects had *MLL* gene rearrangement, two had *Philadelphia-positive* chromosomes, and eight had *FMS-like tyrosine kinase 3 (FLT3-ITD)* internal tandem duplication (FLT3-ITD) with a ratio >0.4. Two subjects died before starting chemotherapy. Ten patients (25%) received acute lymphoblastic leukemia (ALL) induction, and all achieved complete remission (CR) with no induction deaths and no shift of therapy. Thirty patients (75%) started therapy with acute myeloid leukemia (AML) induction: five (16.6%) died during induction, 17 (56.7%) achieved CR, and 10 patients received maintenance ALL therapy after ending AML treatment. Four of the eight patients with induction failure were switched to ALL therapy. The 5-year event-free survival (EFS) and overall survival (OS) rates were 56.7% [standard error (SE): 8.1%] and 61% (SE: 8%), while the cumulative incidence of relapse was 21.7% (SE: 6.7%), with a median follow-up duration of 5.8 years. Patients treated with ALL-directed therapy had a 5-year EFS rate of 111 70% (SE: 14%) and OS rate of 78.8% (SE: 13%). Patients treated with ALL-directed therapy had a 5-year EFS rate of 70% (SE: 14.5%) and OS rate of 78.8% (SE: 13%). *FLT3-ITD* mutation showed a significantly lower 5-year EFS rate of 28.6% (SE: 17%) vs. 75% (SE: 9%) for the wild type, p = 0.032. Undernourished patients with a body mass index (BMI) *z*-score ≤-2 at presentation had a significantly lower 5-year EFS rate of 20% (SE: 17%) compared to 61.8% (SE: 8%) for patients with BMI *z*-score >-2, p = 0.015.

**Conclusion:**

This study supports ALL-directed therapy for pediatric MPAL in a setting of LMIC. Given the poor outcome of *FLT3-ITD*, the role of *FLT3* inhibitor needs to be explored in this subset of cases.

## Introduction

Acute leukemias have historically been classified into lymphoid or myeloid subtypes according to the pattern of surface antigen expression and treated accordingly. However, mixed-phenotype acute leukemia (MPAL) is a form that is featured by either the presence of several lineage-specific markers on a single blast population or the presence of two different lineage blast populations (1).

MPAL has always been intriguing because of its rarity and challenging diagnosis, with a reported incidence of 2%–3% of all acute leukemias. In an effort to define biphenotypic leukemias, the European Group for the Immunological Classification of Leukemias (EGIL) established a system for scoring that included many markers and was very inclusive. The WHO in 2008 proposed a new categorization that is simple and more stringent and depends on the specificity of a few markers to define MPAL; this classification was refined in 2016 (2).

MPAL is challenging because there is no consensus regarding the optimal treatment approach. Only a few publications in pediatrics, primarily retrospective and with a limited number of cases, and the lack of any prospective clinical trial make the decision regarding treatment complicated. Therapeutic options include using acute lymphoblastic leukemia (ALL), acute myeloid leukemia (AML), or a combination of both therapies. Also, transplanting hematopoietic stem cells in initial remission is variable and employed at the decision of the attending physician (3).

Adding to the complexity of pediatric MPAL, the outcome of acute leukemia, in general, in low- and middle-income countries (LMICs) is inferior to that of developed countries, and it varies by country, with cure rates ranging from 40% to 70% (4). Survival rates improve as protocol intensity increases, but therapy-related mortality rates increase as well, necessitating a focus on supportive care and treatment protocol adjustment (5).

This study aimed to report the clinical and genetic characteristics, therapy, and outcome of MPAL cases over a 10-year interval from a large pediatric cancer hospital in a developing country.

## Patients and Methods

The MPAL cases’ computerized medical records were analyzed retrospectively between July 2007 and December 2017 at the Children’s Cancer Hospital Egypt (CCHE-57357). The Scientific and Medical Advisory Committee authorized the study, which was carried out according to Good Clinical Practice guidelines. Patients were included if they were below the age of 18 years and newly diagnosed with MPAL in accordance with the EGIL criteria and then WHO classification 2008/2016. Patients with cytogenetic abnormalities characteristic for ALL (e.g., TCF3/PBX1 or ETV6/RUNX1) or AML (e.g., CBFB/MYH11, PML/RARA, or RUNX1/RUNX1T1) were excluded.

Diagnostic workup for acute leukemia included complete blood cell (CBC) count utilizing Coulter LH 750 cell counter (Coulter Electronics, Hialeah, FL, USA) and bone marrow (BM) extraction, with the assessment of Leishman-stained peripheral blood (PB) and BM smears with cytochemical stains (6). Flow cytometric immunophenotyping was done utilizing a standard array of monoclonal antibodies (MoAbs) by the Navios 6-color flow cytometer (Beckman Coulter, Life Science Division, Indianapolis, IN, USA) for an initial phenotype. A panel of markers done included CD19, CD22, CD79a, and CD10 for B-cell lineage; CD3, CD5, CD7, CD2, Cd4, and CD8 for T-cell lineage; CD13, CD33, CD117, MPO, CD11b, CD64, CD65, CD14, and CD15 for myeloid lineage; and CD34, DR, CD45, CD38, CD56, and Tdt as lineage-nonspecific and stem cell markers.

Metaphase preparations were obtained from BM samples utilizing standardized cytogenetics techniques. In addition, Giemsa staining was performed, whereas clonal chromosomic aberrations were documented following the International System for Human Cytogenetic Nomenclature, while Fluorescence *in situ* hybridization (FISH) was done on the same culture’s metaphase preparations as standard karyotyping. *KMT2A* break-apart probe was utilized based on the instructions of the manufacturer (Metasystems, Germany, and Abbott, USA). Slides were analyzed *via* a Leica DM5500 B microscope and then followed by capturing images *via* an image analyzer system (Applied Imaging Ltd.) and a JAI video camera (7).

Using flanking primers, PCR amplification of the juxtamembrane portion of the *FMS-like tyrosine kinase 3 (FLT3)* gene, specific for internal tandem duplication (ITD) mutations, was performed and size fractionated by capillary electrophoresis. A (*FLT3-ITD*)-to-wild type allelic ratio was calculated and reported for positive patients.

Nutritional status at presentation was assessed by weight for age (WFA), height for age (HFA), and body mass index (BMI) that were classified using standard deviation units referred to as Z scores based on the WHO criterion (8). Patients who were ≤2 standard deviations below the reference median were considered underweight (WFA *z*-score ≤-2), stunted (HFA *z*-score ≤-2), or undernourished (BMI *z*-score ≤-2).

### Treatment

Patients received either AML therapy adopted from the Children’s Oncology Group (COG) treatment protocol (9) or ALL therapy adopted from St. Jude Total Therapy Study XV (10). AML- or ALL-like induction therapy was started based on the BM aspirate morphology and the predominant phenotypic markers. If morphology and markers equally denoted both phenotypes, AML-like induction was the therapy of choice. Patients who started AML-like induction either received four courses of AML therapy only or continued to receive ALL maintenance therapy according to the treating physician’s preference. Subjects with elevated minimal residual disease (MRD) or induction failure were scheduled to have hematopoietic stem cell transplantation (HSCT) consolidation if they had a matching sibling donor. Imatinib was given to patients with *Philadelphia-positive* MPAL.

### Statistical Analysis

Depending on the normality of the distribution, nominal variables were provided as means with standard error (SE) or medians with interquartile ranges. Percentages, frequency distributions, and descriptive statistics were computed for the study subjects’ outcome variables, baseline characteristics, and other factors of interest. Fisher exact test was used for comparison between groups.

For MRD, BM morphology and flow cytometry were used to measure treatment response. Complete remission (CR) is known as fewer than 5% blasts in the marrow of bones, provided that the MRD is also <5% and in the absence of extramedullary disease at the completion of the first induction course.

The Kaplan–Meier method was followed to conduct the survival analysis and then compared *via* the log-rank test, performed with SPSS version 25. Overall survival (OS) was evaluated from the diagnosis date until the date of last contact death. Event-free survival (EFS) was calculated from the diagnosis date until initial failure, including relapse at any site, death during treatment, or date of the last contact for patients who were failure-free at the time of analysis. Survival probabilities were presented as percentages and SEs.

Cumulative incidence of relapse (CIR) was calculated to estimate relapse incidence in a competing risk setting with Grey’s test and was conducted utilizing the R statistical software v.3.2.3. Statistical significance was set at a p-value <0.05.

## Results

### Patient Characteristics

This study included 42 patients (24 boys, 57.1%), with a median age of 9 years (range, 0.8–17.5 years), newly diagnosed with MPAL fulfilling the criteria for EGIL and/or WHO classification 2008/2016. Both criteria for EGIL and WHO 2008/2016 were fulfilled in 35 patients, while six patients fulfilled the EGIL score only, and one case with bilineal disease fulfilled WHO criteria only.

At presentation, the median white blood cell (WBC) count was 43.25 × 10^9^/L (range, 1.8–416.6 × 10^9^/L), with 13 patients (30.9%) presenting with hyperleukocytosis ≥100 × 10^9^/L. Central nervous system (CNS) involvement was detected in four patients (9.5%). At diagnosis, 5 patients (11.9%) were undernourished (BMI *z*-score ≤-2), 7 patients (16.7%) were underweight (WFA *z*-score ≤-2), and 4 patients (9.5%) were stunted (HFA *z*-score ≤-2). The BM morphology was myeloid in 26 patients (61.9%), dual myeloid and lymphoid in 10 (23.8%), lymphoid in four (9.5%), and undifferentiated acute leukemia in two (4.7%). The immunophenotyping was T/Myeloid in 24 patients (57.1%), B/Myeloid in 16 (38.1%), B/T in only one case, and missing in one patient. Two cases were bilineal with two separate leukemic clones, and the rest were biphenotypic. CD34-positive expression, a marker of hematopoietic stem cells, was detected in 32/40 patients (80%).

BM aspirate morphology, cytochemistry, and flow cytometry images of a case with T/Myeloid phenotype are illustrated in **Supplementary Figures S1, S2**.

A normal karyotype was observed in 18 subjects (42.9%), 3 patients (7.1%) had *MLL-r*, 2 patients (4.8%) had *Philadelphia* chromosome t(9,22), while 19 patients (45.2%) had other cytogenetic abnormalities. Complex karyotype, defined as a minimum of three chromosomal abnormalities, with one structural aberration at least, was observed in 3 patients. *FLT3* mutation was tested in 28 patients, 8 (28.5%) were positive for *FLT3-ITD*. All had an allelic ratio >0.4, T/Myeloid phenotype, and no other unfavorable genetic feature. **Supplementary Table S1** summarizes patient characteristics.

### Treatment Details and Outcome

Two patients presented with hyperleukocytosis and intracranial hemorrhage and died shortly before initiating chemotherapy. Of the total 40 patients treated for MPAL, five died during induction chemotherapy, four died in CR, 21 are alive in continuous CR (cCR), seven relapsed, one patient remained refractory, and two were lost to follow-up (FU) on therapy in CR.

**Table 1** summarizes the presenting features and initial therapy. There were no statistically significant differences in the presenting features between MPAL subjects who received an AML or an ALL induction regimen.

**Table 1:** Presenting features and initial therapy.

Patient characteristics ^a^	Total		Initial therapy				p-value
			ALL		AML		
	No.	%	No.	%	No.	%	
	40	100.0	10	25.0	30	75.0	
**Age (years)**							
**<10**	24	60.0	8	80.0	16	53.3	0.263
**≥10**	16	40.0	2	20.0	14	46.7	
**Sex**							
**Boys**	22	55.0	7	70,0	15	50.0	0.464
**Girls**	18	45.0	3	30.0	15	50.0	
**Initial WBC (×10^9^/L)**							
**<100**	29	72.5	7	70.0	22	73.3	1.0
**≥100**	11	27.5	3	30.0	8	26.7	
**CNS involvement at diagnosis** **^b^**							
**Negative**	34	94.4	10	100.0	24	80.0	0.516
**Positive**	2	5.6	0	0.0	2	6.7	
**Nutritional status at diagnosis**							
**BMI *z*-score ≤-2**	5	12.5	1	10.0	4	13.3	1.0
**BMI *z*-score >-2**	35	87.5	9	90.0	26	86.7	
**Phenotype** **^c^**							
**T/M**	22	55.0	5	50.0	17	56.7	0.363
**B/M**	16	40.0	4	40.0	12	40.0	
**B/T**	1	2.5	1	10.0	0	0.0	
**Cytogenetics abnormality**							
** Unfavorable** **^d^**	10	25.0	2	20.0	8	26.7	1.0
**Neutral**	14	35.0	4	40.0	10	33.3	
**None**	16	40.0	4	40.0	12	40.0	
***FLT3-ITD*** **^e^**							
**Wild-type**	20	74.1	3	75.0	17	73.9	0.731
**Mutated**	7	25.9	1	25.0	6	26.1	

ALL, acute lymphoblastic leukemia; AML, acute myeloid leukemia; WBC count, white blood cell count; BMI, body mass index; T/M, T cell/Myeloid; B/M, B cell/Myeloid; B/T, B cell/T cell; FLT3-ITD, FMS-like tyrosine kinase 3 internal tandem duplication.

a This does not include the 2 cases that were not initiated on chemotherapy.

b There were 4 cases with unknown CNS status that were started on AML therapy.

c One case with missing phenotype.

d Unfavorable cytogenetic abnormalities included MLL rearrangement, Philadelphia-positive, monosomy 7, 5q deletion, and complex karyotype.

e FLT3-ITD mutation was tested in 28 cases including one case that did not start chemotherapy.

Ten patients received ALL induction, and 30 patients received AML induction chemotherapy. CR rates after induction were 100% in the ALL cohort and 56.7% (17/30 patients) in the AML cohort. No statistically substantial differences were detected by the end of induction CR rates for patients with either B/Myeloid (10/16; 62.5%) or T/Myeloid (16/22; 72.7%), p = 0.504. The MRD level at the end of induction was <0.1% in 21 patients, ≥0.1% in 12 patients, and not tested in two patients.

### Induction Toxic Mortality

There were five induction deaths due to infectious complications among patients who received AML induction therapy (16.7%), while there were no induction deaths among patients who received ALL induction (p = 0.305).

Analyzing the impact of age and nutritional status at presentation on AML induction toxic mortalities, 2/14 patients ≥10 years old (14.3%) and 3/16 patients <10 years (18.8%) died during induction (p = 0.567). In contrast, there was a tendency toward increased induction deaths among undernourished patients, but the difference was not statistically significant. **Table 2** summarizes the nutritional status at presentation in relation to induction toxic mortality on AML regimen.

**Table 2:** Nutritional status at presentation in relation to induction toxic mortality on AML regimen, overall survival, and event-free survival of all MPAL patients.

Nutritional Status		Induction toxic mortality		p-value	5-year OS ± SE	p-value	5-year EFS ± SE	p-value
		No.	%					
**WFA**	*z*-score ≤-2	2/5	40%	0.183	71.4% ± 17%	0.701	57.1% ± 18%	0.757
	*z*-score >-2	3/25	12%		58.2% ± 19%		56.3% ± 8%	
**HFA**	*z*-score ≤-2	1/2	50%	0.310	50% ± 25%	0.492	50% ± 25%	0.645
	*z*-score >-2	4/28	14.3%		62% ± 8%		57.3% ± 8%	
**BMI**	*z*-score ≤-2	2/4	50%	0.119	40% ± 21%	0.190	20% ± 17%	**0.015**
	*z*-score >-2	3/26	11.5%		63.8% ± 8%		61.8% ± 8%	

WFA, weight for age; HFA, height for age; BMI, body mass index; OS, overall survival; EFS, event-free survival; SE, standard error. Bold p-value indicates a statistically significant difference less than 0.05.

### Postinduction Therapy

All patients who started ALL induction therapy continued the same protocol: seven patients are alive in cCR, two patients relapsed, and one patient with *Philadelphia-positive* B/myeloid MPAL died in CR post-reintensification course.

Of the 17 patients who achieved CR post-AML induction, nine (52.9%) received maintenance ALL therapy after completing the AML protocol, seven are alive in cCR and two were lost to FU during treatment. In contrast, among patients who received AML therapy only, five are alive in cCR, two died in CR, and one relapsed.

Of the eight patients with induction failure, four patients were shifted to ALL protocol (one is alive in cCR, one died in CR, one relapsed, and one remained refractory), while the other four patients continued AML protocol as they achieved CR post-second course (one is alive in cCR and three relapsed).

**Figure 1:**
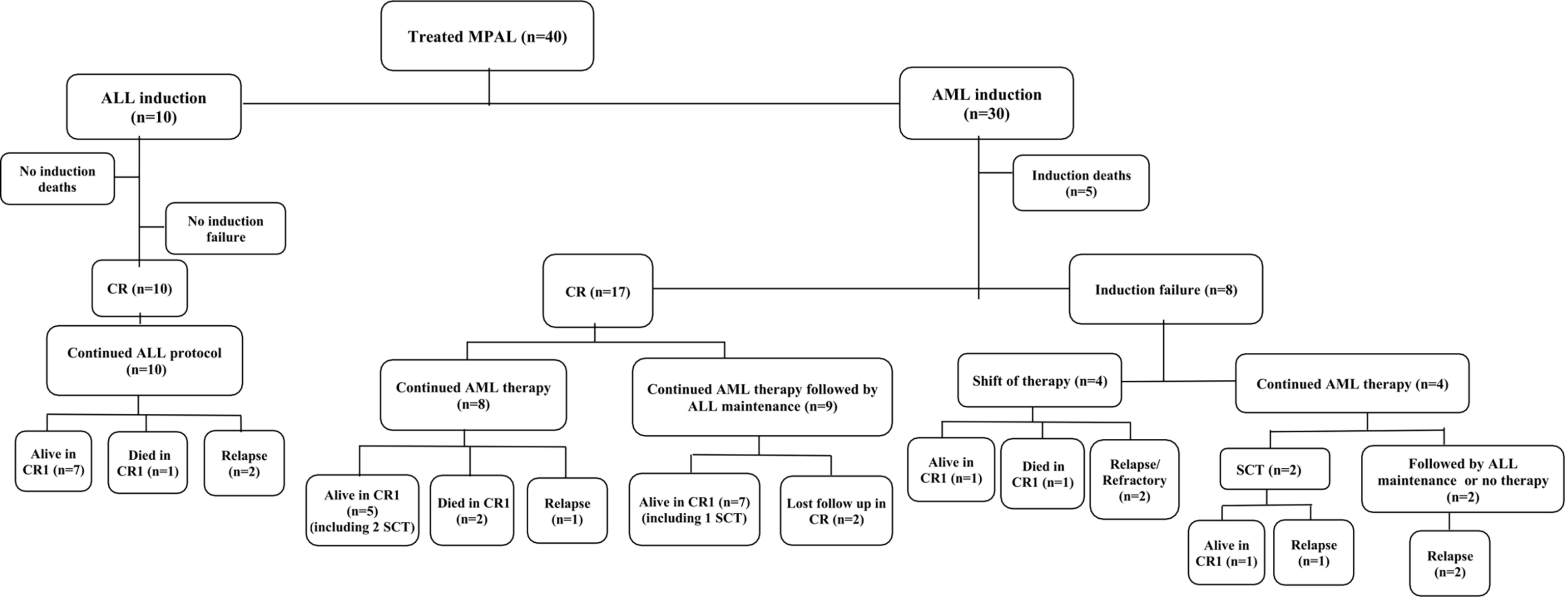
Flow diagram showing the treatment and outcome of the studied patients with mixed-phenotype acute leukemia. Abbreviations: MPAL, mixed-phenotype acute leukemia; n, number; ALL, acute lymphoblastic leukemia; AML, acute myeloid leukemia; CR, complete remission; SCT, stem cell transplant.

Treatment details and outcomes are summarized in **Figure 1** and **Supplementary Table S2**.

### Consolidation With Hematopoietic Stem Cell Transplant

Five patients had consolidation with HSCT, all in first CR. Four patients are alive in cCR, and one died in second CR (relapsed post-HSCT and was salvaged with ALL-like therapy). Patients were selected according to the treating physician’s preference and the presence of a matched sibling donor.

### Survival

**Figure 2:**
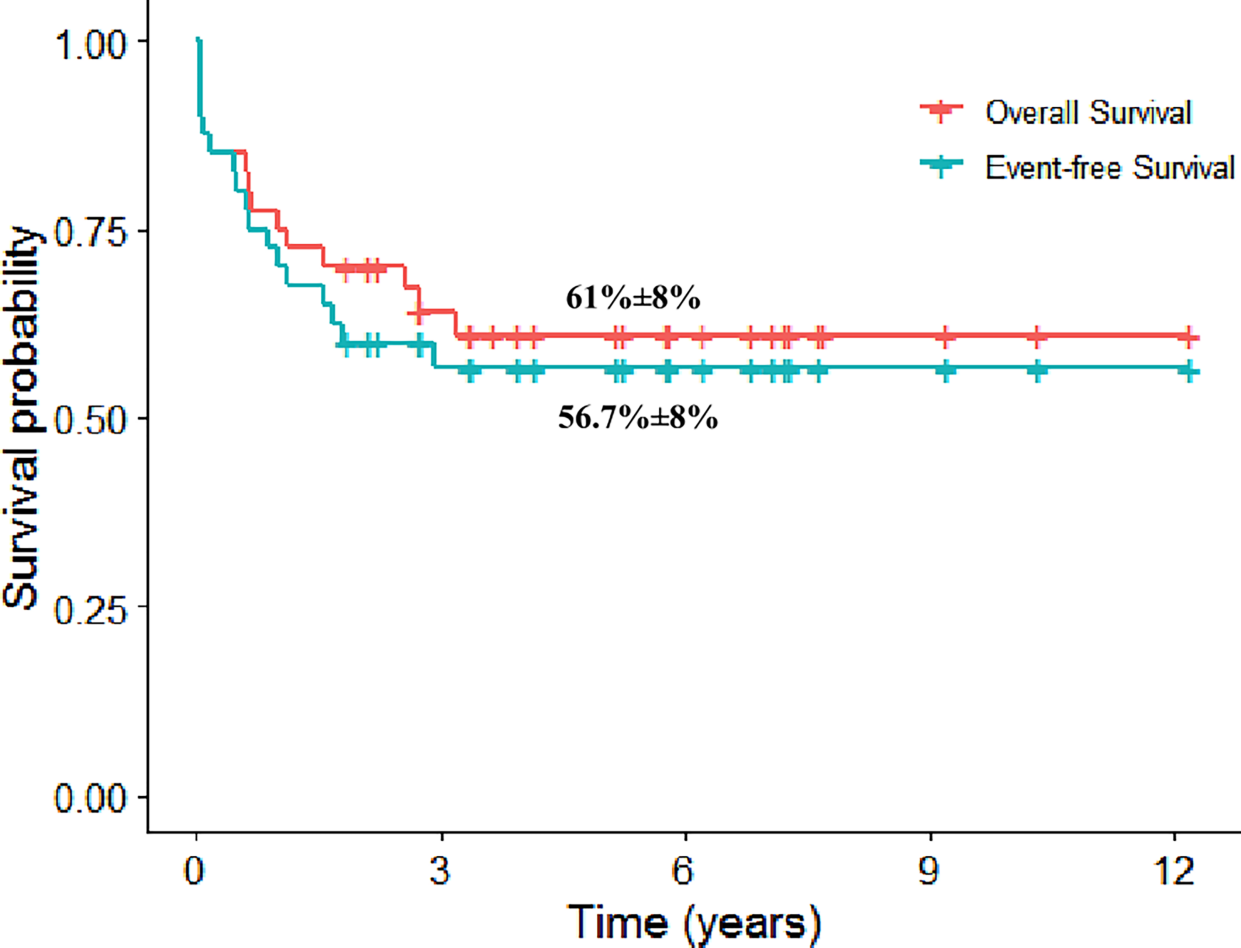
The 5-year Kaplan–Meier plot for overall and event-free survival for patients with mixed-phenotype acute leukemia.

The 5-year event-free survival (EFS) and 107 overall survival (OS) rates were 56.7% [standard error (SE): 8.1%] and 61% (SE: 8%) (**Figure 2**). The cumulative incidence of relapse at 5 years was 21.7% (SE: 6.7%). Patients who are alive in cCR had a median FU of 5.8 years (range, 1.9–12.2 years). It is noteworthy that the 5-year EFS and OS rates on ALL therapy were 70% (SE: 14.5%) and 78.8% (SE:13%).

### Survival in Relation to Prognostic Factors

**Table 3** summarizes the OS, EFS, and CIR rates according to age, sex, presenting WBC count, phenotype, cytogenetic abnormality, *FLT3-ITD* mutation, type of induction chemotherapy, and MRD after induction. No considerable differences were detected in survival rates except for *FLT3* mutation.

**Table 3:** Survival analysis according to studied prognostic factors.

Characteristic		No.	%	5-year OS± SE	p-value	5-year EFS ± SE	p-value	5-year CIR ± SE	p-value
**Age (years)**	≥10	16/40	40%	75% ± 10%	0.237	62.5% ± 12%	0.667	18.8% ± 10%	0.925
	<10	24/40	60%	51.4% ± 10%		52.5% ± 10%		22.1% ± 9%	
**Sex**	Girls	18/40	45%	65.7% ± 11%	0.671	66.7% ± 11%	0.356	16.7% ± 9%	0.704
	Boys	22/40	55%	57.2% ± 11%		48.5% ± 11%		23.7% ± 10%	
**Presenting white blood cell count (×10^9^/L)**	≥100	11/40	27.5%	63.6% ± 14%	0.923	54.5% ± 15%	0.856	18.2% ± 12%	0.789
	<100	29/40	72.5%	59.7% ± 9%		56.9% ± 9%		22.7% ± 8%	
**Phenotype** **^a^**	B/Myeloid	16/38	38%	56.3% ± 12%	0.246	56.3% ± 12%	0.563	12.5% ± 9%	0.339
	T/Myeloid	22/38	57.1%	66.6% ± 10%		57.9% ± 10%		28.1% ± 10%	
**Normal** **karyotype**	No	24/40	60%	65.8% ± 9%	0.571	66.7% ± 9%	0.197	16.7% ± 8%	0.575
	Yes	16/40	40%	55% ± 12%		42.9% ± 12%		25.9% ± 12%	
***FLT3-ITD*** **^b^**	Wild type	20/28	71.4%	75% ± 9%	0.390	75% ± 9%	**0.032**	10% ± 7%	0.066
	Mutated	8/28	28.5%	57% ± 18%		28.6% ± 17%		42.9% ± 22%	
**Induction chemotherapy**	ALL	10/40	25%	78.8% ± 13%	0.191	70% ± 14%	0.361	20% ± 13%	0.937
	AML	30/40	75%	55.4% ± 9%		52.6 ± 9%		20.4% ± 8%	
**MRD post-induction I** **^c^**	<0.1%	21/33	63.6%	80.8% ± 8%	0.061	76.2% ± 9%	0.157	10% ± 7%	0.062
	≥0.1%	12/33	36.4%	44.4% ± 16%		46.7% ± 15%		39.7% ± 15%	

OS, overall survival; EFS, event-free survival; CIR, cumulative incidence of relapse; SE, standard error; FLT3-ITD, FMS-like tyrosine kinase 3 internal tandem duplication; ALL, acute lymphoblastic leukemia; AML, acute myeloid leukemia; MRD, minimal residual disease. Bold p-value indicates a statistically significant difference less than 0.05.

a One case with B/T phenotype and one with missing immunophenotyping not included.

b FLT3-ITD was tested in 28 cases only.

c MRD level were available for 33 patients only.

**Figure 3:**
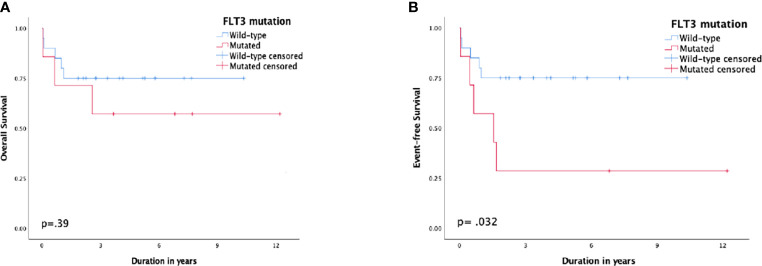
Survival according to FMS-like tyrosine kinase 3 (FLT3) status; **(A)** overall survival, **(B)** event-free survival.

Patients with *FLT3-ITD* had a 5-year EFS of 28.6% (SE: 17%) compared to 75% (SE: 9%) for patients with the wild type, p = 0.032, and the 5-year CIR was 42.9% (SE: 21.7%) vs. 10% (SE: 6.9%), respectively, p = 0.066. Nonetheless, the difference in 5-year OS was not substantial, 57% (SE: 18%) and 75% (SE: 9%), respectively, p = 0.39 (**Figure 3**).

**Figure 4:**
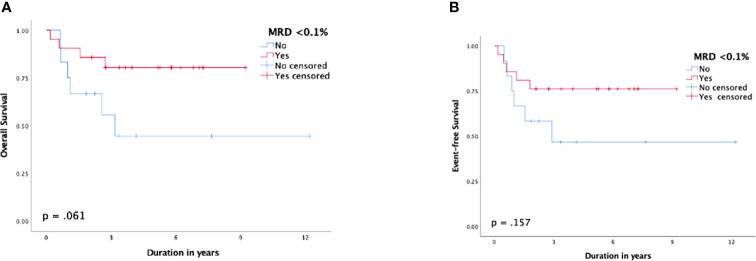
Survival function according to minimal residual disease (MRD) status post-induction; **(A)** overall survival, **(B)** event-free survival.

MRD <0.1% by the end of induction demonstrated a better outcome than those with MRD ≥0.1%, but the difference was not statistically significant. The 5-year OS and EFS rates for patients with MRD <0.1% were 80.8% (SE: 8%) and 76.2% (SE: 9%) compared to 44.4% (SE: 16%) and 46.7% (SE: 15%) for patients with MRD ≥0.1%, respectively (p = 0.061 and p = 0.157, respectively) (**Figure 4**). The 5-year CIR for MRD <0.1% was 10% (SE: 7%) compared to 39.7% (SE: 15%) for MRD ≥0.1%, p = 0.062. Patients who experienced induction failure had a considerably poor outcome, having a 5-year OS rate of 16.7% (SE: 14%).

Undernourished patients with a BMI *z*-score ≤-2 at presentation had a significantly lower 5-year EFS of 20% (SE: 17%) compared to 61.8% (SE: 8%) for patients with a BMI *z*-score >-2, p = 0.015 (**Table 2**).

Both patients with bilineal phenotype were T/myeloid and had a poor outcome. One patient had *monosomy 7* and remained refractory to therapy. The other patient had *del 5q*, relapsed and died in relapse, although he achieved a negative MRD after an ALL-like induction.

## Discussion

MPAL represents an uncommon subtype of acute leukemia. There is still no universal agreement on treatment because of its rarity and complexities. Its pathogenesis might include blocked differentiation of leukemic cells, which cannot be identified or removed by the immune system (1, 11).

According to the initial phenotype of our patients, T/Myeloid was slightly higher than those with B/Myeloid, and only one case had the rare phenotype B/T. Our study showed a slight difference in the incidence, as others reported more cases with B/Myeloid phenotype (12–14). There was no difference in the outcome according to the phenotype with almost identical 5-year EFS, which is in concordance with the results reported in St. Jude’s study (15).

A strong positive CD34 expression, indicative of hematopoietic stem cells, observed in 80% of MPAL cases, may explain why some of these leukemias can differentiate into both lymphoid and myeloid cells (14). The two cases with bilineal disease had a significantly poor prognosis. Previous studies have demonstrated that lower induction remission rates and lineage switch contributed to their poor outcomes (16–19).

The analysis for the clinical features at presentation revealed trends toward subjects with age lower than 10 years, male sex, WBC count lower than 100,000/μl, and T/Myeloid phenotype without any predictor for survival. CNS involvement at diagnosis was observed in 9.5% of our patients. The prevalence of CNS disease in MPAL patients at presentation and relapse needs to be verified in order to have a clear recommendation for CNS-directed therapy (20).

The WHO 2016 classification divided MPAL into *KMT2A rearranged (KMT2Ar), BCR-ABL fusion-positive*, or not otherwise specified (NOS). MPAL patients with *KMT2Ar* tend to present at a younger age, have a B/myeloid phenotype, and have the ability to undergo lineage switch due to retained multilineage potential (21, 22). Our study included three MPAL patients with *KMT2Ar* of them died while receiving induction chemotherapy, and the third case achieved remission on AML therapy and is alive in CR. The influence of cytogenetic aberrations on the outcome could not be determined in relation to the limited number of patients.

Adding the tyrosine kinase inhibitor to a subset of MPAL patients who have *BCR-ABL fusion* has been demonstrated to improve the outcome with acceptable toxicities and is currently the standard of care (23). Our study included two patients with *Philadelphia-positive* MPAL; both achieved remission by adding imatinib to their chemotherapy.

*FLT3-ITD* mutation was positive in 28.5% of the tested patients in our study, and it was associated with a statistically significant inferior EFS. Similar to other studies, it was frequently observed in T/Myeloid phenotype (24, 25). Andrews et al. (26) reported the effective utilization of *FLT3* inhibitors in two cases with *FLT3-mutated* MPAL and supported their use in the treatment of these patients. These inhibitors can also be utilized to treat ZNF384 leukemia (27); nevertheless, prospective randomized trials are scarce.

This type of leukemia expresses markers for B-, T-, and myeloid lineages, which has led the clinician to treat such types of leukemia utilizing approaches that include ALL, AML, or even a hybrid of both therapies, and the use of stem cell transplantation (SCT) was variable between groups. There is no consensus regarding the optimal therapy type, and one could not do so based only on small compiled series with heterogeneous data regarding the type of therapy and the use of HSCT (20).

This ambiguous leukemia has consistently shown worse outcome compared with unambiguous ALL and AML. Most of the data report higher initial remission rates with ALL induction (2, 3, 20, 28, 29). Our study showed a 100% CR rate in patients who were subjected to ALL induction therapy without any induction deaths. In comparison, CR rates following AML induction were only 56.7%, and five patients died of treatment-related toxicities during induction. Even though this difference was not statistically significant, it is more reasonable to choose ALL induction therapy according to the preferable lower toxicity profile and higher CR rates.

In a study by Lohmann et al. (30), age above 10 years was linked to increased toxicity from sepsis and a tendency to have a lower OS during pediatric AML treatment, suggesting that chemotherapeutic drug pharmacokinetics differ in adolescents compared to younger children. Our cohort did not show statistically significant differences in induction mortality with AML regimen and in OS and EFS outcome for this age group.

Undernutrition, a hindering issue in LMICs, increases morbidity and early mortality by causing delayed wound healing, decreased immune function, and increased infectious complications (31). LMICs have much higher rates (40%–90%) of undernutrition than high- or medium-income countries (0%–30%). It is best to assess the nutritional status at the time of diagnosis to aid in treatment planning and therapeutic decisions to avoid occurrence of life-threatening complications (32). In our study, undernourished patients had statistically significant lower 5-year EFS and a tendency to have a higher induction toxic mortality with AML regimen.

In the LMIC setting, using intensive chemotherapy has to be paired with best supportive care that can be further complicated with higher treatment costs and therapy abandonment. The high frequency of toxic deaths, particularly during induction, is also a significant factor contributing to poorer outcomes in developing countries. So, the search for higher remission and lower relapse rates must be balanced against increasing toxic deaths that further justifies ALL induction therapy for patients with MPAL in LMICs (4, 33, 34).

Previous systematic reviews and meta-analyses demonstrated improved survival regarding the ALL approach but were not reproduced in a multivariate analysis (20). In concordance, the 5-year EFS and OS rates on ALL therapy in our study were 70% and 78.8%. We did not compare the differences between ALL and AML regimens on relapse and survival due to the heterogeneity of treatment following AML therapy based on physicians’ preference and selection of some of the survived patients to receive ALL maintenance therapy.

Seetharam et al. (35) reported the outcome of 22 pediatric MPAL treated with lymphoid-directed therapy in a single center in India. The CR rates at the end of induction and 4-year EFS and OS rates were 90%, 60.8%, and 64.9%, respectively, achieving outcomes superior to those seen in AML patients and comparable to those seen in ALL patients.

The COG Acute Leukemia of Ambiguous Lineage Task Force recommended ALL therapy based on the suggested sensitivity due to MPALs’ overlapping biology with ALL, thus avoiding the short- and long-term related toxicity of the AML-like therapy and reserving SCT for patients with high MRD when chemotherapy alone may not be adequate (29). In adults, the approach is to receive HSCT in the first remission (36), while most pediatric MPAL studies do not recommend allogeneic stem cell transplantation (allo-SCT) in the first remission (21, 29, 37, 38). In our study, patients were offered an allo-SCT only if they had a matched sibling donor, as there is no access to unrelated matched donor transplantation in LMICs. Five patients received matched sibling allogeneic stem cell grafts, four of them are alive in remission with an EFS rate of 80%, and consequently, it seems to be an attractive approach, yet with selection bias.

Hybrid therapy was suggested for this type of acute leukemia, since single regimens could lead to clonal or subclonal expansion and eventually to lineage switch (3). However, it was not associated with a higher remission and at the same time harbored toxicities of both treatment arms (20, 28).

MRD testing is a vital tool for risk stratification in patients with ALL or AML. Oberley et al. (38) diagnosed MPAL cases according to the classification of WHO, and patients who were then treated with ALL therapy demonstrated that MRD positivity by the completion of induction had a significantly worse impact on EFS and OS. Our study revealed that MRD <0.1% post-induction tended to have higher OS and lower CIR than MRD ≥0.1% with a marginal p-value of 0.061 and 0.062, respectively. Furthermore, patients with induction failure in this study had poor prognosis with dismal OS. Novel therapies are needed to improve the outcome for such poor responders and in the relapsed/refractory settings. Targeted therapies under investigation include *FLT3* inhibitors and key survival pathway inhibitors such as *B-cell lymphoma 2* (BCL2) inhibitors. Immunotherapies such as blinatumomab and chimeric antigen receptor (CAR) T cell can be considered for patients with CD19-positive MPAL (3).

In conclusion, using ALL treatment for induction demonstrated higher remission rates without toxic mortality in our cohort compared with AML induction. The 5-year EFS rate of 70% on ALL therapy supports lymphoid-directed therapy for these patients especially in LMICs. The retrospective nature of our study and the heterogeneity of treatment are considered limitations. The high incidence of FLT3-ITD and their poor outcome support the rationale for using FLT3 inhibitors to improve the outcome. Future efforts to include targeted therapies based on genetic alterations and phenotypic markers are needed.

## Data Availability Statement

The original contributions presented in the study are included in the article/**Supplementary Material**. Further inquiries can be directed to the corresponding author.

## Author Contributions

MS, IS and SA are guarantors of the integrity of the entire study. AE-H, LL, IS, SA, and MS conceptualized and designed the study. MS explored literature research. MS, IS, SA, and RK analyzed and interpreted the clinical data. SS, NE-S, and SS analyzed and interpreted the laboratory data. AE-N performed the statistical analysis. MS and IS wrote the manuscript and editing was performed by LL and AE-H. IS and AE-H share last authorship. All authors approved the final manuscript, and they are all responsible for all aspects of the work.

## Conflict of Interest

The authors declare that the research was conducted in the absence of any commercial or financial relationships that could be construed as a potential conflict of interest.

## Publisher’s Note

All claims expressed in this article are solely those of the authors and do not necessarily represent those of their affiliated organizations, or those of the publisher, the editors and the reviewers. Any product that may be evaluated in this article, or claim that may be made by its manufacturer, is not guaranteed or endorsed by the publisher.
