# Supplementary material for: Characteristics, Treatment Complexity, and Outcome of Mixed-Phenotype Acute Leukemia in Children in a Low–Middle-Income Country
Source: Front Oncol. 2022 Jul 7;12:941885. doi: 10.3389/fonc.2022.941885 (PMC9300816; doi:10.3389/fonc.2022.941885)

**Supplementary Table S1: Patients characteristics**

| Patient NO. | Age(years)/  Gender | Initial TLC (*10^9^/L) | CNS  status | BMA morphology | Cytogenetics | MPO stain | Myeloid markers | T-lineage markers | B-lineage markers | Phenotype | Bilineal | EGIL | WHO |
| --- | --- | --- | --- | --- | --- | --- | --- | --- | --- | --- | --- | --- | --- |
| #1 | 11.4/F | 135.7 | CNS I | Myeloid | 46, XX [20] | NA | CD13, MPO and CD117 | CD7, CD5, CD2 and Cytoplasmic CD3 |  | T/Myeloid |  | BAL | MPAL |
| #2 | 5.7/M | 78.8 | CNS I | Myeloid | 46, XY[20] | Positive | CD13, MPO and CD117 | CD7, CD2 and Cytoplasmic CD3 |  | T/Myeloid |  | BAL | MPAL |
| #3 | 9.7/F | 329.3 | CNS I | Myeloid | 46, XX [20] | Positive | CD13, CD117 and CD11b | CD7, CD2 and Cytoplasmic CD3 |  | T/Myeloid |  | BAL | MPAL |
| #4 | 15.3/F | 44.6 | CNS III | Myeloid | 46, XX[20] | Positive | CD13,CD33, MPO and CD117 | CD7, CD2 and Cytoplasmic CD3 |  | T/Myeloid |  | BAL | MPAL |
| #5 | 9.8/F | 3.3 | CNS I | AUL | 46, XX [20] | Positive | CD13, CD33 and weak MPO |  | CD19, CD79a, CD22 and CD10 | B/Myeloid |  | BAL | MPAL |
| #6 | 4.1/M | 42.6 | NA | Myeloid | 46, XY[20] | NA |  |  |  | NA |  | BAL |  |
| #7 | 4.7/M | 7.6 | NA | Myeloid | 45,XY -7 [20] | Positive | One population with good expression CD34, DR, dim CD33 and dim CD45 | Another population of abnormal T cell cytoplasmic CD3, CD5, CD1, CD4, CD8, CD2 and CD7 (T intermediate phenotype) |  | T/Myeloid | Bilineal | BAL | MPAL |
| #8 | 9.1/F | 312.5 | CNS I | Lymphoid | 46,XX del(12)(p12) [15], 46,XX [5] | Negative | CD33, CD13, CD117 and dim CD133 |  | CD19, CD22, CD79a and TdT | B/Myeloid |  | BAL |  |
| #9 | 15.2/F | 90.8 | CNS I | Myeloid | 45, XX, -7, t(9;22)(q34;q11)[20] | Positive | CD13, CD33 and CD117 |  | CD19, CD79a, CD10 and TdT | B/Myeloid |  | BAL | MPAL |
| #10 | 11.1/M | 119 | CNS I | Myeloid | 46, XY[20] | Positive | CD13, dim 33, CD123, CD117and CD11b | CD7, CD2, cytoplasmic CD3 and TdT |  | T/Myeloid |  | BAL | MPAL |
| #11 | 16.0/M | 2.7 | CNS I | Lymphoid | 55,XY, +8, +10, +11, +12, +12, +19, +19, +21, +21 | Negative | MPO |  | CD22, CD79a, CD10, Cytoplasmic u and very dim CD19 | B/Myeloid |  | BAL | MPAL |
| #12 | 15.0/F | 100 | NA | Myeloid | 46, XX [20] | Positive | CD13 and CD117 | CD7, CD2 and Cytoplasmic CD3 |  | T/Myeloid |  | BAL | MPAL |
| #13 | 8.8/M | 19 | CNS I | Myeloid | 46, XY[20] | Positive | CD13, MPO and CD117 | CD7, CD2 and Cytoplasmic CD3 |  | T/Myeloid |  | BAL | MPAL |
| #14 | 8.8/M | 11.7 | CNS I | Myeloid | 46, XY[20] | Positive | CD33 |  | CD19, CD79a, CD22 and TdT | B/Myeloid |  | BAL | MPAL |
| #15 | 4.7/M | 389.2 | NA | Myeloid | 46, XY[20] | Positive | CD117 and CD71 | CD7, cytoplasmic CD3, CD2, dim CD5 and TdT |  | T/Myeloid |  | BAL | MPAL |
| #16 | 4.5/M | 8 | CNS I | AUL | 46, XY, ins(2;?)(p12;?), t(4;?)(q31;?), del(5)(q23), -16, -9, +20, +mar[14] / 46, XY[8] | Negative | CD33, CD11b, CD65, CD64 and CD14 | Cytoplasmic CD3, CD7, CD5 and CD2 |  | T/Myeloid |  | BAL | MPAL |
| #17 | 11.3/M | 270 | CNS I | Lymphoid | 46, XY[20] | Negative |  | sCD3, CD5 and CD7 | CD19 and CD79a | B/T |  | BAL | MPAL |
| #18 | 3.9/M | 3.7 | CNS I | Myeloid | 46, XY, del(9)(q22)[5] / 92, XXYY, inc[3] | Positive | MPO, lysozyme and CD117 | CD3, scattered TdT, CD7 and CD5 |  | T/Myeloid |  | BAL | MPAL |
| #19 | 0.9/F | 380 | CNS I | Dimorphic | 47, XX, t(3;11;?)(q21;q23;?), +8[20] | Negative | CD33, CD64, CD11c, CD11b, dim CD13 and CD14 |  | CD19, CD22, CD79a, TdT and dim CD10 | B/Myeloid |  | BAL | MPAL |
| #20 | 16.7/M | 2.7 | CNS I | Dimorphic | 94, XXYY, +4, -17, -17, +20, +21, +21[4] / 46, XY[16] | Positive | Dim CD33, dim MPO(23%), CD11c and CD11b |  | CD22, CD19, CD79a, dim Cytoplasmic u and CD20(12%) | B/Myeloid |  | BAL | MPAL |
| #21 | 13.5/F | 189 | CNS I | Myeloid | 46, XX, del(16)(q13)[20] | Positive | CD13 and CD117 | Dim cytoplasmic CD3, CD7 and CD2 |  | T/Myeloid |  | BAL | MPAL |
| #22 | 8.7/M | 416.6 | CNS III | Dimorphic | 46, XY[20] | Positive | Dim CD13 and CD117 | Cytoplasmic CD3, CD7, CD2 and TdT |  | T/Myeloid |  | BAL | MPAL |
| #23 | 12.8/F | 3.2 | CNS I | Dimorphic | 46, XX, del(1)(p32), del(5)(q33)[15] / 46, XX[5] | Positive | CD33 and CD11b | CD7, cytoplasmic CD3, dim CD5 and TdT |  | T/Myeloid |  | BAL | MPAL |
| #24 | 2.8/M | 4.7 | CNS I | Lymphoid | 46, XY, del(5)(q13)[11] / 46, XY[14] | Negative | One population CD34, CD7, CD33 and DR | Another population CD7, CD1, CD5, dim CD2, CD4, CD8, cytoplasmic CD3, CD7 and bright CD45 |  | T/Myeloid | Bilineal |  | MPAL |
| #25 | 2.8/F | 4.3 | CNS I | Myeloid | 47, XX, +8[17] / 46, XX[3] | Positive | CD33, CD13, CD117, CD65, CD15, CD71, strong MPO, dim CD11c and CD11b |  | CD79a, Cytoplasmic u, and strong CD19 | B/Myeloid |  | BAL | MPAL |
| #26 | 5.5/M | 22.3 | CNS I | Myeloid | 47, XY, +X[18] / 46, XY[2] | Positive | CD117, CD71 and dim CD133 | Dim CD4, CD7, and cytoplasmic CD3 is positive in 36% of this population |  | T/Myeloid |  | BAL | MPAL |
| #27 | 15.4/F | 31 | CNS I | Myeloid | 46, XX, t(4;11)(q21;q23)[20] | Positive | CD33, MPO, Cytoplasmic CD13, CD133, dim CD11c and dim CD11b |  | CD19 and CD79a | B/Myeloid |  | BAL | MPAL |
| #28 | 17.5/M | 43.9 | CNS I | Dimorphic | 46, XY[20] | Positive | CD13, CD117, dim CD11b and weak MPO(17%) | Cytoplasmic CD3, CD7 and CD2 |  | T/Myeloid |  | BAL | MPAL |
| #29 | 4.4/M | 104.9 | CNS I | Dimorphic | 51, XY, +8, +10, +14, +20, +22[10]/ 46, XY[10] | Negative | CD33, dim CD64, dim CD11b, CD65 and CD66 | CD4, CD2, dim CD5 and CD7 |  | T/Myeloid |  | BAL |  |
| #30 | 7.9/M | 247.9 | CNS I | Myeloid | 46, XY, add(9)(p21)[7]/ 46, XY[13] | Positive | CD13, CD117, dim CD11b and MPO (on 12 - 14% of the malignant clone with 2 - 5% on the same CD34+ve cells expressing cCD3) | Cytoplasmic CD3 good expression (on all malignant clone CD34+ve), bright CD7, CD2, and CD1a (on 15 % of the malignant clone) |  | T/Myeloid |  | BAL | MPAL |
| #31 | 12.3/M | 305.8 | CNS III | Myeloid | 46, XY[20] | Positive | CD13, CD117, dim CD11b and dim MPO (on 15 - 16% of the malignant clone) | Cytoplasmic CD3 moderate to good expression (on all malignant clone CD34+ve), CD7, CD2 and CD5 |  | T/Myeloid |  | BAL | MPAL |
| #32 | 9.8/M | 65.1 | CNS I | Dimorphic | 45, XY,-7,t(9;22)(q34;q11)[20] | Positive | CD13 |  | CD19, CD79a, bright CD10 and dim CD22 | B/Myeloid |  | BAL | MPAL |
| #33 | 14.7/F | 56.9 | CNS I | Myeloid | 46, XX, nuc ish(5' MLLx2)(3' MLLx1), (5'MLL con 3'MLLx1)[198/200] | Positive | CD33, dim CD13, CD117 and dim CD65 |  | CD19, CD79a and dim CD22 | B/Myeloid |  | BAL | MPAL |
| #34 | 1.4/M | 23 | CNS III | Dimorphic | 47, XY,t(1;12)(p36;p13),del 7(q22),+19[20] | Positive | CD117, dim CD33, CD13, CD56, CD71 and dim CD11b | CD7, dim weak CD5 and CD4 |  | T/Myeloid |  | BAL |  |
| #35 | 12.8/M | 19.3 | CNS I | Myeloid | 46,XY[20] | Positive | CD13, CD11b, CD117, dim CD33, CD65, heterogenous CD11cand MPO | CD7, CD2 and Cytoplasmic CD3 (on 20.3%) |  | T/Myeloid |  | BAL | MPAL |
| #36 | 1.6/F | 9.4 | CNS I | Myeloid | 46, XX[20] | Positive | MPO, CD13, CD33, CD117, CD11b, CD65 and CD71 |  | CD19 and CD79a | B/Myeloid |  | BAL | MPAL |
| #37 | 7.2/M | 14.7 | CNS I | Dimorphic | 46, XY, t(11;12)(p15;p13)[18]/46, XY[2] | Positive | CD117, CD33, MPO, dim CD11b, and CD71 | Cytoplasmic CD3, CD7 and very dim CD2 |  | T/Myeloid |  | BAL | MPAL |
| #38 | 7.9/F | 3.2 | CNS I | Myeloid | 46, XX, t(2;?!3)(p16;q12)[18]/46, XX[2] | Positive | CD117, CD13, DR, MPO, dim CD11b, and CD33 | Cytoplasmic CD3, CD7 and CD2 |  | T/Myeloid |  | BAL | MPAL |
| #39 | 0.8/F | 90.1 | CNS I | Myeloid | 50, XX, +add(8)(q24), +21, +21, +mar[12]/46, XX[8] | Negative | CD33, cytoplasmic CD13, CD117 and CD71 |  | Dim CD19 and CD22 | B/Myeloid |  | BAL |  |
| #40 | 3.4/M | 87.7 | CNS I | Dimorphic | 46, XY[20] | Positive | CD33, Weak cCD13 and weak MPO (6%) |  | CD19, CD79a and CD22 | B/Myeloid |  | BAL | MPAL |
| #41 | 11.7/F | 2.8 | CNS I | Myeloid | 46, XX, t(4;11)(q21,q23)[5] | Positive | CD33, CD13, CD117, MPO and dim CD71 |  | CD19 and dim CD22 | B/Myeloid |  | BAL | MPAL |
| #42 | 10.7/F | 1.8 | CNS I | Myeloid | 47, XX, +8, del(9)(q22)[12]/47,XX, +8, del(9)(q22), del(11)(q22)[4]/46, XX[4] | Positive | CD13, dim CD33, CD117, MPO, CD71, CD65, CD14 and dim CD4 |  | Dim CD19 and dim weak CD79 | B/Myeloid |  | BAL |  |

**AUL (Acute undifferentiated leukemia), BAL (Biphenotypic acute leukemia), CNS (Central nervous system), MPAL (Mixed phenotype acute leukemia), MPO (Myeloperioxidase), NA (not available)**

**Supplementary Table S2: Treatment and outcome**

| Patient No. | Induction treatment type | Outcome of induction | Post-induction therapy | Treatment Outcome | Survival Status |
| --- | --- | --- | --- | --- | --- |
| #1 | AML | Induction failure | Shifted to ALL therapy | cCR | Alive |
| #2 | AML | Induction failure | Shifted to ALL therapy | Died in CR | Dead |
| #3 | AML | Induction failure | AML total of 4 courses + HSCT | Relapse | Dead |
| #4 | AML | CR | AML total of 4 courses | cCR | Alive |
| #5 | ALL | CR | ALL | cCR | Alive |
| #6 | AML | Died induction | --- | Died induction | Dead |
| #7 | AML | Induction failure | Shifted to ALL therapy | Died refractory | Dead |
| #8 | ALL | CR | ALL | cCR | Alive |
| #9 | AML | Induction failure | Shifted to FLA/M + ALL maintenance | Relapse | Dead |
| #10 | AML | CR | AML total of 4 courses | Relapse | Alive |
| #11 | AML | CR | AML total of 4 courses | cCR | Alive |
| #12 | AML | CR | AML total of 4 courses | Died in CR | Dead |
| #13 | AML | Induction failure | AML total of 4 courses + ALL maintenance | Relapse | Dead |
| #14 | AML | CR | AML total of 4 courses | Died in CR | Dead |
| #15 | AML | Died induction | --- | Died induction | Dead |
| #16 | AML | CR | AML total of 4 courses + ALL maintenance | cCR | Alive |
| #17 | ALL | CR | ALL | cCR | Alive |
| #18 | AML | CR | AML total of 4 courses + ALL maintenance | Lost follow up in CR |  |
| #19 | AML | Died induction | --- | Died induction | Dead |
| #20 | AML | Died induction | --- | Died induction | Dead |
| #21 | AML | CR | AML total of 4 courses + HSCT | cCR | Alive |
| #22 | Steroid only | NE | --- | Died disease not evaluated | Dead |
| #23 | AML | CR | AML total of 4 courses + HSCT | cCR | Alive |
| #24 | ALL | CR | ALL | Relapse | Dead |
| #25 | AML | CR | AML total of 4 courses + ALL maintenance | cCR | Alive |
| #26 | AML | CR | AML total of 4 courses + ALL maintenance | cCR | Alive |
| #27 | AML | Died induction | --- | Died induction | Dead |
| #28 | AML | CR | AML total of 4 courses + ALL maintenance | Lost follow up in CR |  |
| #29 | AML | CR | AML total of 4 courses + ALL maintenance | cCR | Alive |
| #30 | ALL | CR | ALL | cCR | Alive |
| #31 | Steroid only | NE | --- | Died disease not evaluated | Dead |
| #32 | ALL | CR | ALL | Died in CR | Dead |
| #33 | AML | CR | AML total of 4 courses + ALL maintenance + HSCT | cCR | Alive |
| #34 | AML | CR | AML total of 4 courses + ALL maintenance | cCR | Alive |
| #35 | ALL | CR | ALL | Relapse | Alive |
| #36 | AML | CR | AML total of 4 courses + ALL maintenance | cCR | Alive |
| #37 | ALL | CR | ALL | cCR | Alive |
| #38 | ALL | CR | ALL | cCR | Alive |
| #39 | AML | Induction failure | AML total of 4 courses | Relapse | Dead |
| #40 | ALL | CR | ALL | cCR | Alive |
| #41 | AML | CR | AML total of 4 courses | cCR | Alive |
| #42 | AML | Induction failure | AML total of 4 courses + HSCT | cCR | Alive |

**ALL (Acute lymphoblastic leukemia), AML (Acute myeloid leukemia), CR (Complete remission), cCR (continuous complete remission), HSCT (Hematopoietic stem Cell Transplant), NE (Non-evaluable)**

**Supplementary Figure S1:** Bone marrow aspirate morphology and cytochemistry images

(a)Bone marrow aspirate showing infiltration with blast cells which were large, heterogenous with fine nuclear chromatin, multiple nucleoli and relatively abundant cytoplasm. (b) Positive Myeloperoxidase with occasional Auer rods. (c) Acid phosphatase showed intense polar positivity.

a) b) c)


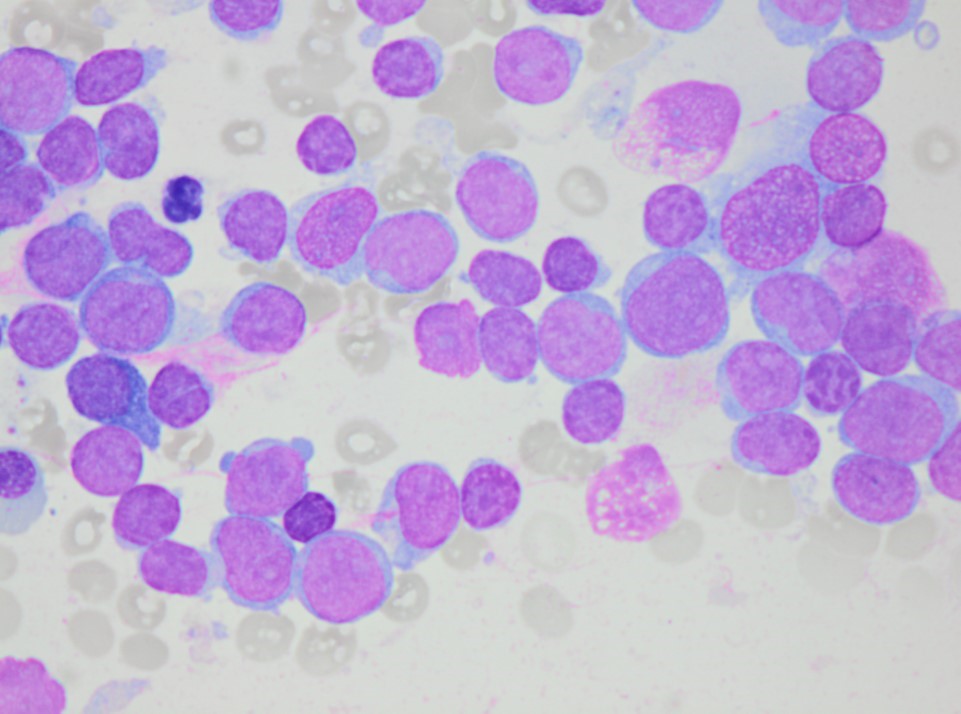

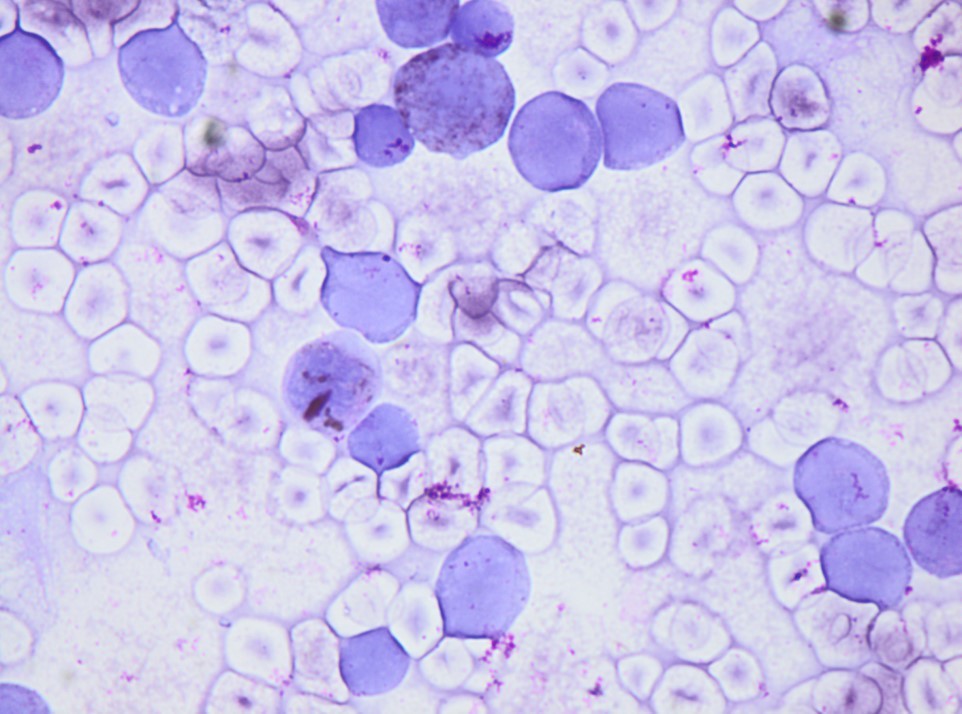

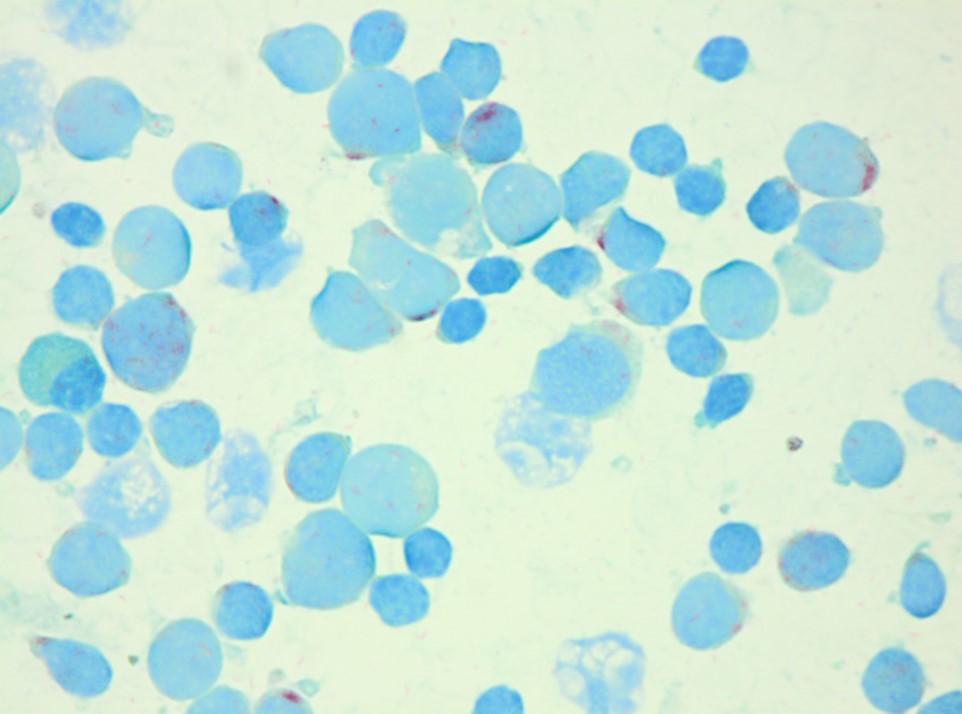


**Supplementary Figure S2:** Flow cytometry images showing co-expression of T cell markers (cytoplasmic CD3, CD5 and CD7) with stem cell marker(CD34), and myeloid markers (MPO and CD33)


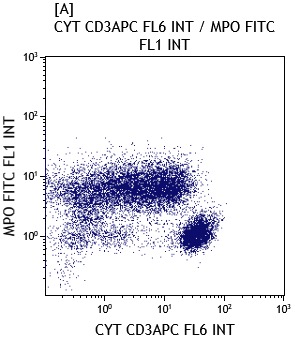

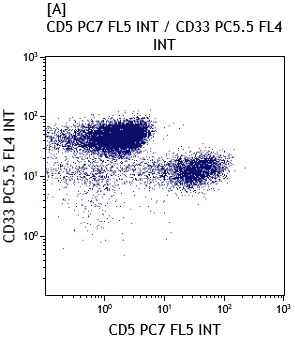

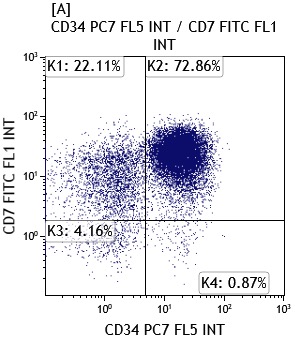

Supplement: Supplementary file 1 [file DataSheet_1.docx]
